# Removal of Acetic Acid from Bacterial Culture Media by Adsorption onto a Two-Component Composite Polymer Gel

**DOI:** 10.3390/gels8030154

**Published:** 2022-03-02

**Authors:** Junya Kato, Takehiko Gotoh, Yutaka Nakashimada

**Affiliations:** 1 Graduate School of Integrated Sciences for Life, Hiroshima University, 1-3-1 Kagamiyama, Higashihiroshima 739-8530, Hiroshima, Japan; junyakato@hiroshima-u.ac.jp; 2 Graduate School of Advanced Science and Engineering, Hiroshima University, 1-4-1 Kagamiyama, Higashihiroshima 739-8527, Hiroshima, Japan

**Keywords:** polymer gel, cationic gel, adsorption, organic acid, acetic acid, fermentation, culture medium

## Abstract

Organic acids, including acetic acid, are the metabolic products of many microorganisms. Acetic acid is a target product useful in the fermentation process. However, acetic acid has an inhibitory effect on microorganisms and limits fermentation. Thus, it would be beneficial to recover the acid from the culture medium. However, conventional recovery processes are expensive and environmentally unfriendly. Here, we report the use of a two-component hydrogel to adsorb dissociated and undissociated acetic acid from the culture medium. The Langmuir model revealed the maximum adsorption amount to be 44.8 mg acetic acid/g of dry gel at neutral pH value. The adsorption capacity was similar to that of an ion-exchange resin. In addition, the hydrogel maintained its adsorption capability in a culture medium comprising complex components, whereas the ion-exchange did not adsorb in this medium. The adsorbed acetic acid was readily desorbed using a solution containing a high salt concentration. Thus, the recovered acetic acid can be utilized for subsequent processes, and the gel-treated fermentation broth can be reused for the next round of fermentation. Use of this hydrogel may prove to be a more sustainable downstream process to recover biosynthesized acetic acid.

## 1. Introduction

Microbial fermentation processes have been widely employed for the production of various chemicals. Microbial fermentation uses biomass as a carbon-neutral resource instead of fossil resources or organic waste, thus providing a promising solution for reducing global carbon emission. Furthermore, some microorganisms can utilize carbon dioxide (CO_2_) and convert it into valuable chemicals [1,2,3,4,5,6,7]. 

Organic acids are produced as metabolites during microbial fermentation. Short-chain volatile fatty acids, especially acetic acid, are common microbial metabolites. They are useful as bulk chemicals in industries and have broad applications in food and pharmaceutical industries [1,2,5,6,8]. Acetic acid has an annual global demand of 10 million tons. Microbial fermentation products currently cover ~10% of the market, and it is anticipated that the coverage will increase because of low CO_2_ emission. Among the fermentation processes, the use of acetogens, a group of microorganisms, is advantageous and efficient strategy. The metabolic pathway of acetogens involves the fixation and conversion of CO_2_ to acetic acid using electrons generated from sugar metabolism or using hydrogen (H_2_). Acetogens can convert sugars into acetic acid without liberating CO_2_ or even utilize CO_2_ depending on the energy received from H_2_ [9,10].

Microbial fermentation is challenged by downstream processes for recovering the biosynthetic products, and acetic acid is no exception. Because acetic acid is released into the culture medium in a dilute form and is soluble in an aqueous medium, separation of the acid is difficult. The separation processes, which mostly involve the use of organic solvents, are expensive and have adverse effects on the environment. Nonetheless, alamine/diisobutyl ketone extraction has been shown to reduce global warming potential and fossil fuel usage [6]. Distillation does not require organic solvents but is limited to relatively low-volatile-temperature chemicals. Azeotropic distillation must be used to increase the concentration of acetic acid. However, this process is usually applied for acetic acid concentrations higher than that obtained in fermentation, is energy-intensive, and requires further development [11,12]. Product separation is vital for achieving the purity and concentration required in subsequent industrial processes and for obtaining high productivity and titer in the fermentation process. Fermentation products often have inhibitory effects on the metabolism of microorganisms, thereby limiting the fermentation titer. Acetic acid has also been reported to inhibit microbial activity, and the inhibitory effect originates from disrupted intracellular homeostasis, and not just by a low pH [13]. This inhibitory effect has also been observed for other organic acids, such as propionic acid [8,14]. It is important to remove acetic acid to maintain the microbial performance. 

In this study, we attempted to separate acetic acid from the fermentation culture medium using a hydrogel polymer. Hydrogel polymers have attracted attention over the years because of their reasonable capacity and selectivity for removing hazardous materials, such as metals from industrial wastewater [15,16,17,18]. Because fermentation culture media change properties, such as the pH, throughout the fermentation process and have complex components, treatment with a hydrogel polymer could facilitate the removal of the desired component. We also tested whether the removed acetic acid, which is harmful for microorganisms, can be recovered using a simple method as a valuable material for industrial applications. The hydrogel-treated medium was examined for its reuse as a culture medium. The hydrogel itself could be easily separated from the culture medium. Therefore, this study can contribute to the development of a process with low environmental burden. 

## 2. Results and Discussion

### 2.1. Concept of Two-Component Copolymer Gel for Acetic Acid Adsorption

**Figure 1:**
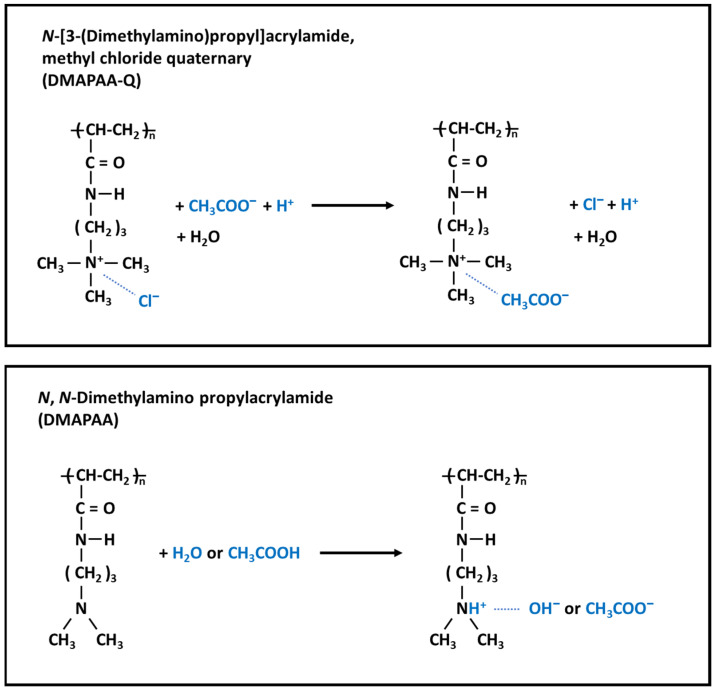
Chemical structures of two types of acrylamide-based polymers in the AQ11 copolymer gel. DMAPAA-Q is enriched with chloride ions (Cl^−^). DMAPAA is enriched with a water molecule when acetic acid is absent. The plausible interactions are shown.

Acetic acid is a carboxylic acid with two carbon atoms and a p*K*_a_ value of 4.8. Depending on the pH, it exists in the ionized or undissociated form in an aqueous medium. We aimed to capture both the forms of acetic acid from the culture medium over a range of pH, including the pH at which acid production lowers the pH. Two types of acrylamide monomers were selected to capture each form: *N*-[3-(dimethylamino)propyl]acrylamide, methyl chloride quaternary (DMAPAA-Q) and *N*,*N*-dimethylamino propylacrylamide (DMAPAA). DMAPAA-Q served as an adsorbent to capture the dissociated acetate ions, and DMAPAA served as an adsorbent to capture the undissociated acetic acid and dissociated protons (Figure 1). A copolymer with equal amounts of these two monomers was synthesized and is referred to as AQ11.

### 2.2. Basic Characteristic of Acetic Acid Adsorption on AQ11

**Figure 2:**
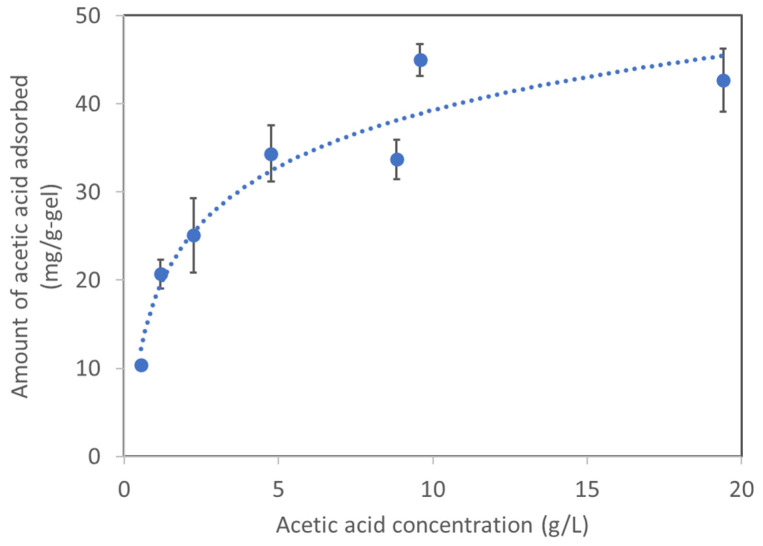
Adsorption isotherm of AQ11 against various concentrations of acetic acid at pH 7.0.

We tested the adsorption of acetic acid on the AQ11 gel at various concentrations of acetic acid (mainly acetate) solution at pH 7.0. The solution primarily contained acetic acid; the sodium ions present in the medium were derived from pH adjustment. We examined the basic characteristics at neutral pH because this pH is maintained in most of the controlled fermentation processes. The AQ11 gel adsorbed acetic acid, and the corresponding adsorption isotherm (Figure 2) revealed the saturation of adsorption at acetic acid concentrations between 10 and 20 g/L. Because the saturation concentration was below that required for microbial growth inhibition (~48 g/L), as reported for the acetogenic bacterium, *Moorella thermoacetica* [13], the AQ11 gel would exhibit maximum capacity when the acetic acid is to be recovered in practice. Fit of the data (R^2^ = 0.98) to the Langmuir equation (Equation (1)) suggested that the maximum acetic acid adsorbed by AQ11 was 44.8 mg/g of gel. K_b_ was calculated as 0.91 (Figure S1). The fit is consistent with that reported in previous studies on the adsorption of arsenic by DMAPAA-Q [15,19].

C_e_/Q_e_ = 1/(K_b_ Q_max_) + C_e_/Q_max_ (1)

Here, C_e_ is the concentration of acetic acid (g/L) at equilibrium, Q_e_ is the adsorption capacity of the AQ11 gel (mg/g-gel), K_b_ is the adsorption coefficient, and Q_max_ is the maximum adsorption amount. 

**Figure 3:**
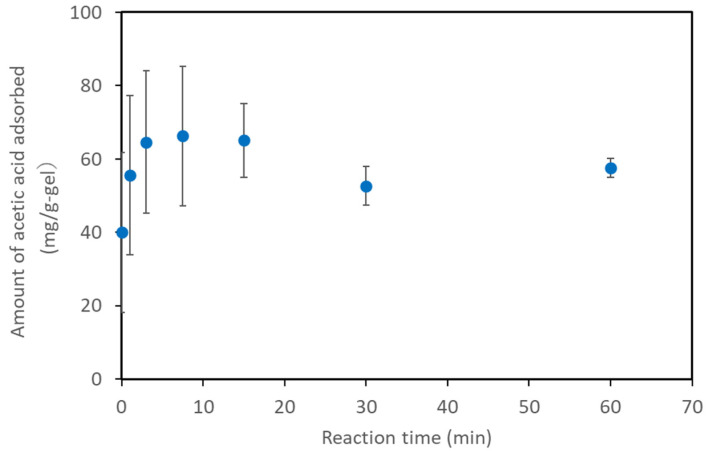
Relationship between the reaction time and amount adsorbed.

Next, we measured the time required to reach the adsorption equilibrium at an acetic acid concentration of 10 g/L. Although we tested the basic adsorption characteristics through overnight reactions, in practical use, the reaction time is a key factor when employing polymer gels for acetic acid removal. The experimental setup was the same as that shown in Figure 2. However, the solution in which AQ11 was immersed was sampled at various time points, and the acetic acid concentration was measured (Figure 3). The acetic acid concentration decreased immediately after the gel was added, and the adsorption initially increased over time. Up to 30 min, the amount adsorbed was more than the saturation amount, with significant variation among the samples collected at different time points. This may indicate non-specific binding to the gel up to 30 min. After 30 min, the adsorbed amount decreased, and the adsorption was near saturation. This trend indicates that a certain time period is required for stable and specific adsorption. Stable adsorption requires approximately 30 min, and this reaction time will be reasonably short for practical use. 

### 2.3. Comparison of AQ11 with Other Adsorbents

**Figure 4:**
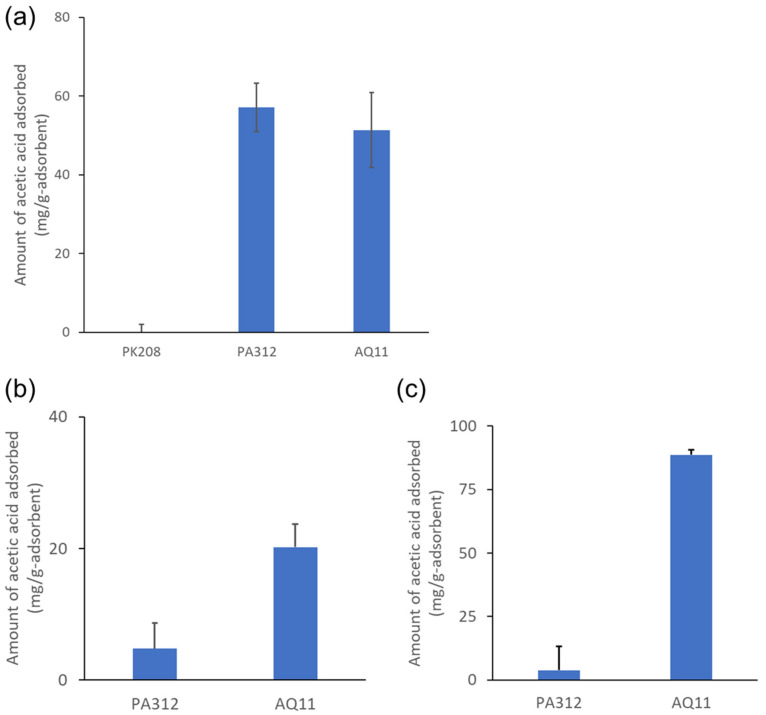
Comparison of adsorption by AQ11 and ion exchange resins. (**a**) Comparison of PK208, PA312, and AQ11 in a 10 g/L acetic acid solution at pH 7.0. (**b**) Comparison of PA312 and AQ11 in a culture medium. (**c**) Comparison of PA312 and AQ11 in an acetic acid solution (10 g/L) without adjusting the pH (the pH of the solution was 2.9).

We compared the adsorption capacity of some selected adsorbents to determine whether AQ11 is superior to other adsorbents. We selected cation exchange and anion exchange resins for comparison: PK208 is a strongly acidic cation exchange resin, and PA312 is a strongly basic anion exchange resin. As expected, PK208 did not adsorb acetic acid because acetate ions are negatively charged and do not bind to cation exchange resins. On the other hand, the adsorption on PA312 was 12% higher than that on AQ11 in a 10 g/L acetic acid solution (pH 7.0; Figure 4a). The acetic acid adsorption on PA312 was consistent with the product data sheet [20], which indicates 1.2 meq/mL of the adsorbent of the minimum salt splitting capacity; at least 40 mg of acetic acid can be captured by one gram of the PA312 resin, considering 1 g of the resin was equivalent to 1.8 mL in the assay solution. The use of ion-exchange resins is one of the most common adsorption methods for organic acid recovery [21]. However, in practice, organic acids, including acetic acid, are recovered from solutions that have inconsistent properties. The target solution, the microbial culture broth, has complex properties with complex components and various pH values. Some of the attempts to recover acetic acid using ion exchange resins have not been completely successful because of the presence of impurities, and the pretreatment steps required [22]. Therefore, we examined the adsorption under various conditions. 

### 2.4. Adsorption Capability of AQ11 in a Culture Medium and at Various pH Values

We first examined the adsorption in a culture medium for selectivity. We tested the culture medium for the acetogenic bacterium, *Acetobacterium woodii*. Acetic acid (10 g/L) was added to a fresh culture medium to compare the adsorption capacities of PA312 and AQ11. In this case, PA312 exhibited almost no adsorption, whereas AQ11 maintained its adsorption capability (Figure 4b). Although acetic acid was the main component in this culture medium, the adsorption of acetic acid on PA312 was inhibited by the components in the culture medium. Thus, AQ11 was superior to PA312 in terms of selectivity in solutions containing impurities, such as a bacterial culture medium. 

We also evaluated the binding capacity at lower pH. At a lower pH, acetic acid exists in an undissociated form, and the ionic interactions required for adsorption are absent. The DMAPAA component of the AQ11 polymer can capture undissociated acetic acid (Figure 1). We tested the adsorption capacity in an acetic acid solution without adjusting the pH (Figure 4c). PA312 showed almost no binding, as predicted, whereas the adsorption on AQ11 was nearly two-fold higher than that at neutral pH. Thus, AQ11 can tolerate low pH values for adsorbing acetic acid.

**Figure 5:**
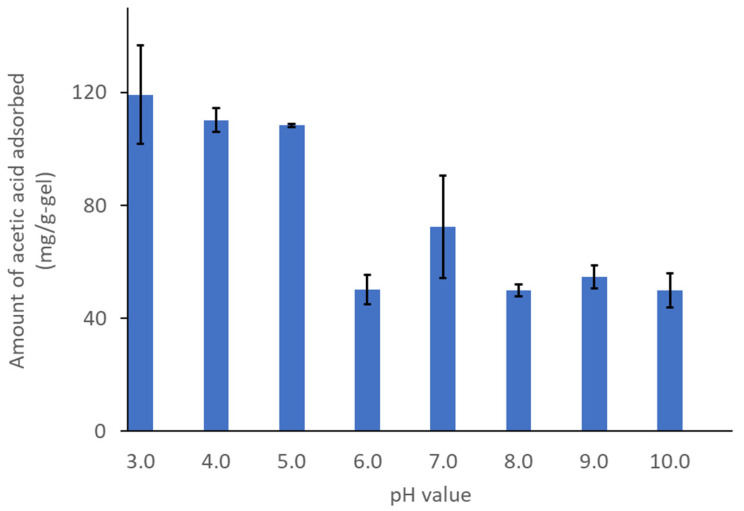
Comparison of the adsorption in acetic acid (10 g/L) at various pH.

Furthermore, AQ11 maintained its adsorption capacity at various pH values (Figure 5). The adsorption was tested from pH 3.0 to 10.0, with one unit increment in pH. Interestingly, high adsorption was observed between pH 3.0 and 5.0. Adsorption was also observed in the pH range of 6.0–10.0, although it was lower than that at low pH values. This is consistent with the chemical nature of acetic acid, which has a p*K*_a_ value of 4.8. AQ11 shows higher adsorption toward acetic acid at a low pH; more than 50% of the acetic acid exists in the undissociated form between pH 3.0 and 5.0, whereas a major proportion (>90%) of the acid was in the dissociated form between pH 6.0 and 10.0. DMAPAA-Q and DMAPAA adsorbed the dissociated acetate ions and undissociated acetic acid under the former condition, whereas DMAPAA-Q mainly adsorbed the dissociated acetate ions under the latter condition. The pH-dependent adsorption can be attributed to the difference in the adsorption capacities of DMAPAA-Q and DMAPAA. 

### 2.5. Recovery of Acetic Acid from AQ11

**Figure 6:**
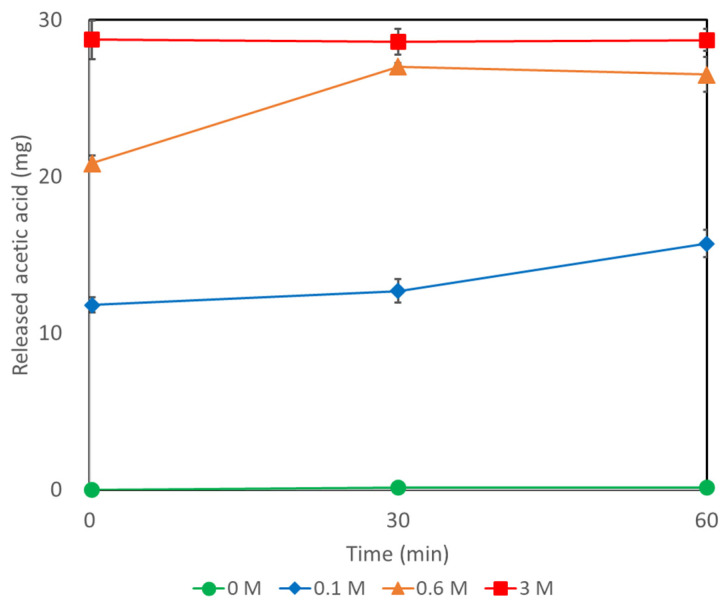
Desorption of the adsorbed acetic acid from AQ11 in NaCl solutions.

The recovery of acetic acid from the gel is necessary to utilize the adsorbed acetic acid for industrial applications. The recovery process must be rapid and involve only simple treatment steps. We used a sodium chloride (NaCl) solution to desorb the acetic acid from the gel. The AQ11 gel with adsorbed acetic acid was extensively washed with water before the desorption experiment. The gel was then immersed in water and in 0.1, 0.6, and 3.0 M NaCl solution (Figure 6). While no desorption of acetic acid was observed in water, acetic acid was desorbed and hence, detected in the NaCl solutions. We sampled the solutions over time and found that acetic acid was desorbed in the NaCl solution in a dose-dependent manner and that the desorbed amount increased over time. This trend was apparent in the case of 0.6 M NaCl; the maximum release was observed at 30 min. Among all three NaCl concentrations, the maximum release upon immersion of the gel was immediately observed in the 3.0 M NaCl solution. Complete release was not observed after 1 h in 0.1 M NaCl. The released acetic acid in the 0.1 M NaCl solution was measured after two days; yet the value increased slightly and did not reach the maximum. Therefore, we concluded that a high concentration of NaCl (3.0 M) or incubation for half an hour in a 0.6 M NaCl solution was sufficient for the desorption of the adsorbed acetic acid. 

Thus far, desorption after adsorption has been challenging because a considerable amount of acid/alkaline agents or alcohols is required [23,24]. Our study demonstrates an environmentally friendly, simple, and short-term method for the desorption of acetic acid. The recovered acetic acid is suitable for biological processes, such as fermentation using marine bacteria [25]. The NaCl concentration used for desorption (0.6 M) was close to the NaCl concentration in seawater. 

### 2.6. Recycling of AQ11-Treated Culture Broth for Fermentation

We tested whether the culture medium after AQ11 treatment could be recycled for fermentation. If AQ11 has no harmful effects on the microbial activity during fermentation, recycling can render the process more sustainable with less water consumption. As a test case, we examined the fermentation of acetic acid from sugar using *A. woodii*. The culture broth to be tested was prepared by growing *A. woodii* in a medium supplemented with 10 g/L fructose, followed by treatment with AQ11 after complete conversion of fructose to acetic acid. The bacterial cells were removed by centrifugation and filtration before AQ11 treatment. A fermentation experiment was then conducted by using the recycled medium instead of a part of water to prepare the culture medium. As shown in Table 1, the culture with the recycled medium showed comparable conversion of the substrate to the product. Moreover, AQ11 treatment did not have harmful effects on the microbial activity. 

### 2.7. Adsorption of Short-Chain Organic Acids by AQ11

Finally, we assessed the possibility of applying AQ11 to separate other short-chain organic acids. Organic acids with different chain lengths, namely, formic acid (C1), acetic acid (C2), propionic acid (C3), and butyric acid (C4), were selected. Each acid solution was prepared at concentrations between 160 and 200 mM, and the pH was adjusted to 7.0. The organic acids were adsorbed onto AQ11 either with similar adsorption capacity or with a slightly higher capacity (up to 30% more molecules) (Table 2). Thus, the AQ11 gel can also be used to separate different types of organic acids. These organic acids are in a group of volatile fatty acids (VFAs) produced by fermentation and are useful in industries [26].

## 3. Conclusions

This study shows that the two-component composite copolymer AQ11 effectively separates and recovers acetic acid from a microbial culture medium. AQ11 is superior to ion-exchange resins in selectively capturing acetic acid with a high capacity from an aqueous medium containing complex components. The recovery of acetic acid by desorption requires a simple process and a short reaction time, which are suitable for industrial applications. Furthermore, the treated medium can be recycled for subsequent culturing. Thus, the use of AQ11 may serve as a sustainable post-fermentation method. 

## 4. Materials and Methods

### 4.1. Materials

Monomeric DMAPAA-Q and DMAPAA were obtained from KJ Chemicals Corporation (Tokyo, Japan). Potassium dichromate (K_2_Cr_2_O_7_) and *N*,*N*,*N*′,*N*′-tetramethylethylenediamine (TEMED) were obtained from Nacalai Tesque, Inc. (Kyoto, Japan). *N*,*N*′-methylenebisacrylamide (MBAA) and ammonium peroxodisulfate (APS) were obtained from Sigma Aldrich Co. (St. Louis, MO, USA). All reagents were used as received. Aqueous solutions were prepared using distilled water. PK208 and PA312 were purchased from Mitsubishi Chemical Corporation (Tokyo, Japan). Organic acids were purchased from Nacalai Tesque, Inc. (Kyoto, Japan). *A. woodii* is a type strain DSM 1030.

### 4.2. Synthesis of Hydrogel Adsorbent

In a 30 mL volumetric flask, 3.617 g of DMAPAA-Q (monomer), 1.841 g of DMAPAA (monomer), 0.2698 g of MBAA (cross-linking agent), and 0.08135 g of TEMED (accelerator) were dissolved in distilled water. APS (initiator, 0.15973 g) was dissolved in distilled water in a 5 mL volumetric flask (Table 3). Nitrogen gas (N_2_) was purged for 30 min in each solution to remove oxygen (O_2_) and prevent the inhibition of radical polymerization in the flask containing distilled water. After N_2_ purging, the initiator and monomer solutions were mixed and stirred for 20 s and then charged into a Teflon pipe with an inner diameter of 6 mm. The DMAPAA-Q, DMAPAA, and MBAA were polymerized at 25 °C for 6 h. After polymerization, the gel was removed from the pipe and cut into pieces of 6 mm length. The gel was washed with methanol for 24 h using a Soxhlet extractor (Asahi Glassplant Inc., Arao City, Japan) to remove the unreacted monomers. After washing, the gel was dried at 25 °C for several days and then thoroughly dried in an oven at 50 °C. After drying, the gel was a solid and crushed. Pieces with diameters of approximately 1 mm were selected to perform the adsorption studies. The solid form becomes a gel, once it is immersed in aqueous solutions.

### 4.3. Analytical Methods

The organic acid and fructose concentrations were measured by high performance liquid chromatography (HPLC). The chromatograph (LC-2000 Plus HPLC; Jasco, Tokyo, Japan) equipped with a refractive index detector (RI-2031 Plus; Jasco), Shodex RSpak KC-811 column (Showa Denko, Kanagawa, Japan), and Shodex RSpak KC-G guard column (Showa Denko). The column temperature during the analysis was 60 °C. Ultrapure water containing 0.1% (*v*/*v*) phosphoric acid was used as the mobile phase at a flow rate of 0.7 mL/min. Crotonic acid was used as the internal standard [27]. The experiments were performed in triplicate, and the standard deviations are shown.

### 4.4. Adsorption Assay

In the adsorption experiment, 0.5 g or 1.0 g of the AQ11 gel was dispensed into a 50 mL tube. The pH-controlled organic acid solution was prepared using the organic acid to be tested, sodium salt of organic acid, and NaOH. Sodium formate, sodium acetate, sodium propionate, or sodium butyrate was dissolved in water, and the pH was adjusted by using the corresponding organic acid or NaOH, respectively. For acidic pH to test the adsorption of acetic acid, acetic acid was dissolved in water and the pH was adjusted by NaOH. Adsorption was initiated by adding 20 mL of the corresponding organic acid solution to each tube and shaking it at 180 rpm in a shaker at 30 °C. The mixture was incubated overnight (for 20–24 h) to establish equilibrium unless otherwise noted. The organic acid concentration was measured using HPLC before and after the adsorption. The amount of adsorbed organic acid was calculated using the following equation:

Q = (C_0_ − C_v_) × V × *M*/m (2)

Here, Q is the amount of organic acid adsorbed (g/g-adsorbent), C_0_ is the initial concentration of organic acid (mol/L), C_v_ is the equilibrium concentration or the concentration after adsorption reaction, V is the volume of organic acid solution used for the reaction, m is the mass of adsorbent (g) in a dry form, and *M* is the molar mass of organic acid. 

### 4.5. Desorption Assay

Desorption of acetic acid or acetate ions adsorbed onto AQ11 gel was studied in NaCl solutions. The acetic acid solution, with pH adjusted to 7.0, was removed by centrifugation at 2000 rpm after adsorption. The gel was washed with an equal amount of deionized water to that of the acetic acid solution, separated, and removed by centrifugation. The gel was then washed three times. An NaCl solution, whose volume was the same as that of the acetic acid solution, was then added to the gel to initiate desorption. One milliliter of NaCl solution was sampled over time, and the concentration of acetic acid was measured by HPLC. The amount of acetic acid desorbed was calculated using the following equation:

Q′ = C′_v_ × V′ × M/m (3)

Here, Q′ is the amount of acetic acid released (g acetic acid/g gel), C′_v_ is the concentration of acetic acid in the NaCl solution, V′ is the volume of the NaCl solution at the time of sampling, m is the mass of adsorbent (g), and M is the molar mass of acetic acid.

### 4.6. Fermentation Experiment

The culture medium for growing *A. woodii* was prepared following the protocol for the acetogen *Moorella thermoacetica*; however, the concentration of NaHCO_3_ was different [28]. An NaHCO_3_ stock solution (100 g/L) was prepared and added to achieve a final concentration of 10 g/L. Briefly, a modified ATCC 1754 PETC medium [29] was prepared for the cultures as follows: the basal medium contained: 10.0 g/L fructose, 2.0 g/L yeast extract, 1.2 g/L l-cysteine hydrochloride monohydrate, 1.0 g/L NH_4_Cl, 0.1 g/L KCl, 0.2 g/L MgSO_4_·7H_2_O, 0.8 g/L NaCl, 0.1 g/L KH_2_PO_4_, 0.02 g/L CaCl_2_·2H_2_O, 10.0 g/L NaHCO_3_, 10 mL of trace elements, 10 mL of Wolfe’s vitamin solution [30], and 1.0 mg of resazurin/L of deionized water. The medium was prepared, and *A. woodii* was grown anaerobically [31]. *A. woodii* was grown in 125 mL glass vials at 30 °C in 50 mL of the culture medium dispensed. Fructose consumption and acetic acid production were monitored by sampling 1 mL of the culture, followed by HPLC analysis. To reuse the culture broth after AQ11 treatment, 10 g of the gel was used to remove acetic acid from one vial of the culture broth (~45 mL). The gel was then removed by filtration and centrifugation. The clear broth was sterilized by filtration, followed by N_2_ purging to remove O_2_. The recycled medium was prepared by supplementing with the broth instead of a portion of water (10% of the total volume of the medium). 

## 5. Patents

This work has been included in a patent application by Hiroshima University.

## Figures and Tables

**Table 1:** Comparison of the fermentation profile of *A. woodii* using a fresh medium and a reused medium after treatment with AQ11.

Medium Composite	Fructose Consumption (mM)	Acetic Acid Production (mM)
Fresh medium	57.4 ± 0.4	108.1 ± 3.2
Recycled medium	56.5 ± 1.1	111.6 ± 3.1

**Table 2:** Comparison of the adsorption on the AQ11 gel toward various organic acids.

Organic Acid	Tested Concentration (mM)	Amount Adsorbed (mmol/g-gel)
Formic acid	160	0.88 ± 0.07
Acetic acid	162	0.67 ± 0.08
Propionic acid	178	0.75 ± 0.03
Butyric acid	193	0.80 ± 0.09

**Table 3:** Synthesis condition of the AQ11 hydrogel.

Component Name	Component Type	Molecular Weight	mol/m^3^	g
DMAPAA	monomer	105.22	500	1.841
DMAPAA-Q	monomer	206.71	500	3.617
MBAA	linker	154.17	50	0.2698
TEMED	accelerator	116.21	20	0.08135
APS	Initiator	228.19	20	0.15973

## Data Availability

Electronic supporting information is available.

## Supplementary Materials

The following supporting information can be downloaded at: https://www.mdpi.com/article/10.3390/gels8030154/s1, Figure S1: Langmuir adsorption isotherm plot for the acetic acid adsorption by AQ11.
