# Supplementary material for: Removal of Acetic Acid from Bacterial Culture Media by Adsorption onto a Two-Component Composite Polymer Gel
Source: Gels. 2022 Mar 2;8(3):154. doi: 10.3390/gels8030154 (PMC8950367; doi:10.3390/gels8030154)
Supplement: Supplementary file 1 [file gels-08-00154-s001.zip › gels-1590167-supplementary.pdf]

gels

MDPI

Article

Removal of Acetic Acid from Bacterial Culture Media by Adsorption onto a Two-Component Composite Polymer Gel

Junya Kato [1], Takehiko Gotoh [2,*] and Yutaka Nakashimada [1,*]

Supporting Information

Langmuir plot for determination of maximum adsorption amount and adsorption coefficient

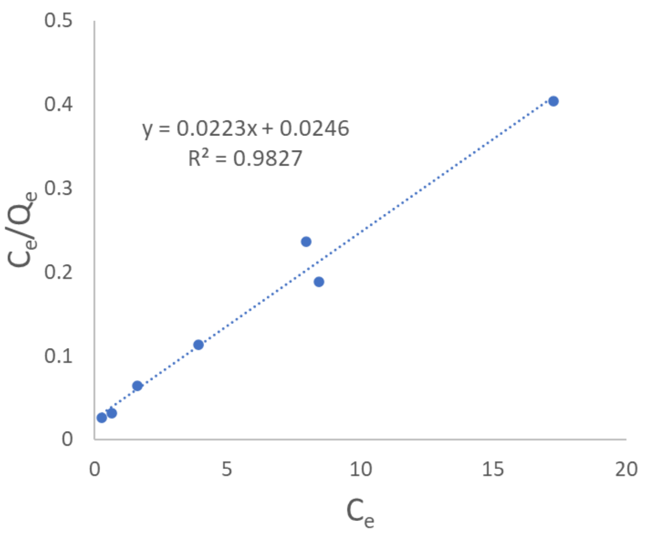

Figure S1. Langmuir adsorption isotherm plot for the acetic acid adsorption by AQ11.

The obtained data for C_e and C_e/Q_e were plotted as shown in Figure S1 (C_e, g/L; Q_e, mg/g gel). The linear approximation formula was also represented with an R^2. Then the Langmuir model equation (1) can be shown as (1′):

$$C_e/Q_e = 0.0223{*}C_e + 0.0246 \quad (1')$$

Therefore, $1/Q_{max} = 0.0223$ and $1/(K_b\ Q_{max}) = 0.0246$ were used to calculate Q_{max} and K_b.
